# Deep learning enhanced transmembranous electromyography in the diagnosis of sleep apnea

**DOI:** 10.1186/s12868-024-00913-9

**Published:** 2024-12-31

**Authors:** Ross Mandeville, Hooman Sedghamiz, Perry Mansfield, Geoffrey Sheean, Chris Studer, Derrick Cordice, Ghodsieh Ghanbari, Atul Malhotra, Shamim Nemati, Jejo Koola

**Affiliations:** 1 Powell Mansfield, Inc., San Diego, CA USA; 2 Division of Biomedical Informatics, Department of Medicine, University of California San Diego, San Diego, CA USA; 3 Department of Neurosurgery, Massachusetts General Hospital, Harvard Medical School, Boston, MA USA; 4 Division of Pulmonary, Critical Care, Sleep Medicine & Physiology, Department of Medicine, University of California San Diego, San Diego, CA United States; 5 Division of Hospital Medicine, Department of Medicine, University of California San Diego, San Diego, CA USA

**Keywords:** Deep learning in medicine, Audio spectral transformer, Sleep apnea diagnosis, Quantitative electromyography, Transmembranous EMG

## Abstract

Obstructive sleep apnea (OSA) is widespread, under-recognized, and under-treated, impacting the health and quality of life for millions. The current gold standard for sleep apnea testing is based on the in-lab sleep study, which is costly, cumbersome, not readily available and represents a well-known roadblock to managing this huge societal burden. Assessment of neuromuscular function involved in the upper airway using electromyography (EMG) has shown potential to characterize and diagnose sleep apnea, while the development of transmembranous electromyography (tmEMG), a painless surface probe, has made this opportunity practical and highly feasible. However, experience and ability to interpret electrical signals from the upper airway are scarce, and much of the pertinent information within the signal is likely difficult to detect visually. To overcome this issue, we explored the use of transformers, a deep learning (DL) model architecture with attention mechanisms, to model tmEMG data and distinguish between electromyographic signals from a cohort of control, neurogenic, and sleep apnea patients. Our approach involved three strategies to train a generalizable model on a relatively small dataset including, (1) transfer learning using an audio spectral transformer (AST), (2) the use of 6,000 simulated EMG recordings, converted to spectrograms and using standard backpropagation for fine-tuning, and (3) application of regularization to prevent overfitting and enhance generalizability. This DL approach was tested using 177 transoral EMG recordings from a prior study’s database that included six healthy controls, five moderate to severe OSA patients, and five amyotrophic lateral sclerosis (ALS) patients with evidence of bulbar involvement (neurogenic injury). Sensitivity and specificity for classifying neurogenic cases from controls were 98% and 73%, respectively, while classifying OSA from controls were 88% and 64%, respectively. Notably, by averaging the predicted probabilities of each segment for individual patients, the model correctly classified up to 82% of control and OSA patients. These results not only suggest a potential to diagnose OSA patients accurately, but also to identify OSA endotypes that involve neuromuscular pathology, which has major implications for clinical management, patient outcomes, and research.

## Introduction

Obstructive sleep apnea (OSA) is a common and serious disorder that affects millions of people in the U.S. and around the world [1]. Sleep apnea is defined by repeated interruptions in breathing during sleep, which can lead to daytime sleepiness, impaired cognitive function, and increased risk of cardiovascular and metabolic diseases [2]. OSA also imposes an important economic burden on individuals, employers, health care systems, and society, with undiagnosed OSA costing the U.S. approximately $149.6 billion (about $460 per person) [3]. Treating OSA improves quality of life and health, as well as reduces the economic costs associated with the disorder [4, 5].

The polysomnogram (PSG) is the current gold standard test for OSA, yielding the apnea-hypopnea index (AHI) that measures respiratory events per hour of sleep as a function of disease severity [6]. PSG has limited access, especially in rural and economically disadvantaged areas, and is costly, cumbersome, and inconvenient, impacting accuracy [7]. Home sleep apnea testing (HSAT) addresses some drawbacks but lacks electroencephalography (EEG) and sleep stage assessment [8]. Both PSG and HSAT lack the versatility to act as an on-demand diagnostic screening tool in hospitals for pre-sedation assessment, as is often desired to ensure the safe use of anesthetics and narcotics. Current standard sleep testing, regardless of venue, may be influenced by physiological variables and does not typically provide insights regarding pathophysiological mechanisms. Accurate detection of OSA presence, severity, and endotype is crucial for ensuring appropriate therapy, which to date can include behavior modification, oral devices, surgery, hypoglossal nerve stimulation, neuromuscular stimulation, or positive airway pressure [9]. Therefore, there exists an important need for an alternative, simpler, and less invasive method for OSA detection, quantification, and characterization [10]; in particular, one that can be implemented for point-of-care screening and diagnosis across a broad spectrum of clinical contexts.

Electromyography (EMG) in OSA has been the subject of only limited investigation to date. However, a few prior studies [11–15] have suggested potentially useful differences in the EMG signals recorded from the genioglossus in those with and without OSA. Evidence of local nerve and muscle pathologies, as well as neurogenic EMG changes, has been found in patients with OSA who show no other clinical signs of a neuromuscular disorder [16–21]. In addition to neuromuscular pathology, alterations in signal that do not result in classic quantitative electromyographic abnormalities, such as resting activity levels and changes in motor recruitment throughout the respiratory cycle, are likely important [13–15]. Several limitations including the invasive nature of testing have resulted in little advance in this potentially fruitful diagnostic avenue. A major step toward realizing the diagnostic ability of EMG in OSA has recently been achieved by overcoming the invasive and painful nature of testing; a novel mucous membrane surface probe has been developed termed transmembranous EMG (tmEMG™) that can record electrical activity from intraoral and oropharyngeal muscles with similar diagnostic quality to needle EMG [22]. However, due to the scarcity of experience and skill in performing and analyzing EMG data from this area, and our hypothesis that much of the pertinent diagnostic information within the signal is likely difficult to detect visually, we aimed to develop and evaluate a novel Deep Learning (DL) method to enhance the diagnostic ability of tmEMG in OSA.

In this study, we hypothesized that transformers [23] are capable of modelling tmEMG data effectively. Transformers are a type of DL model architecture that use attention mechanisms to learn from sequential data. Almost all state-of-the-art modern methods for audio signal processing and natural language processing tasks (such as automated transcription, machine translation, text summarization, and question answering, among others) are based on the transformers model architecture. In this work, we used a transformer-based model to encode tmEMG signals into latent vectors which were used for downstream classification of tmEMG recordings into control vs. neurogenic or control vs. OSA classes. Where possible, we adhere to the CLAIMS checklist as a guide for reporting the findings from our study [24].

## Methods

### Data

Data for this study were obtained from a prior pilot study published by our group [22] that demonstrated the quality of tmEMG signals closely matched that of standard invasive needle EMG (nEMG). Please refer to the original study for the full description of the patient population, characteristics, and study design. Briefly, the pilot study was a prospective cohort study with blinded data analysis involving six healthy participants, five patients with moderate to severe OSA, and five patients with amyotrophic lateral sclerosis (ALS) who showed clinical signs of bulbar involvement. Each patient underwent sampling from bilateral palatoglossus (PG) and genioglossus (GG), using both tmEMG and needle EMG with band-pass filter settings at 10 Hz to 10 kHz and sampling frequency at 64 kHz. ALS patient EMG data was classified as “neurogenic”, meaning injury to the peripheral nerve, by dual subject-matter expert review. Including both tmEMG and nEMG, and recordings from both GG and PG muscles, a total of 177 labelled EMG recordings of 10 s in duration were de-identified and exported for use in assessing the DL model methodology of the current study. Prior to use in training and validation of the DL model, manual data selection was performed with EMG recordings cropped to remove extraneous signal that did not include the target EMG signal; a separate DL model is being developed to automate this quality assurance process. This process of manual cropping resulted in EMG recordings being of variable length, up to 10 s in duration. Down-sampling was performed to conform with sampling requirements for the AST (16 kHz) and to reduce data size.

### Transformers

We used transformers, a DL model architecture with attention mechanisms, to model EMG data. Transformers are integral to state-of-the-art methods in audio signal and natural language processing tasks. The introduction of the Transformer model by Vaswani et al. [25] marked a departure from conventional sequence transduction approaches, which heavily relied on recurrent neural networks (RNNs) and convolutional neural networks (CNNs). This novel architecture utilizes attention mechanisms exclusively, discarding the use of RNNs and CNNs, leading to significant gains in parallelization and training efficiency. Notably impactful in machine translation, the Transformer sets new benchmarks with commendable results, including scores of 28.4 BLEU (Bilingual Evaluation Understudy) on the WMT (Workshop on Machine Translation) 2014 English-to-German and a record-breaking 41.8 BLEU on the WMT 2014 English-to-French translation tasks.

At its foundation, the Transformer is built with an encoder and decoder, each composed of layers that employ self-attention and fully connected networks. The encoder concurrently processes input sequences, while dependencies between the input and output are delineated by the self-attention mechanism. The decoder, empowered by its own self-attention and additional encoder-decoder attention mechanisms, produces the output sequence and selectively concentrates on pertinent segments of the input. Positional information is integrated into input embeddings via positional encodings. The multi-head attention aspect of this architecture allows for simultaneous focus on various subdivisions of the sequence, thereby covering extensive dependency ranges. With these innovations, the architecture provides a boost in training speed and enhancement of performance on translation tasks, proving the efficacy of attention-centric models in sequence-related issues.

Extending the principles of the Transformer architecture to visual recognition, Dosovitskiy et al. [26]

presented the Vision Transformer (ViT), which applies this model directly to images for classification. ViT represents a deviation from the CNN-dominated image recognition field; it partitions images into patches, linearly embeds them, combines with positional embeddings, and processes them through a traditional Transformer encoder. When trained on expansive datasets, ViT delivers impressive results across multiple image recognition benchmarks, outshining contemporary CNNs while also requiring fewer computational efforts for training. Through this innovation, the ViT contends the prevalent dependence on CNNs and promotes Transformers as capable and scalable alternatives for image classification.

In the audio realm, the Audio Spectrogram Transformer (AST) [23], a model eschewing convolutions in favor of pure attention-based mechanisms, explores the viability of non-CNN architectures for audio classification. The AST applies directly to audio spectrograms and discerns long-range context from the earliest layers of the network. Mirroring ViT architectural methodology, AST excels on various audio classification benchmarks such as AudioSet, ESC-50, and Speech Commands V2, surpassing previous models and showcasing its versatility as a universal audio classifier. With a straightforward structure and rapid convergence rates during training, AST offers a compelling alternative to conventional CNN-based models in audio analysis.

Audio signals and certain physiological signals, such as EMG, share numerous similarities. Both are sampled at very high frequencies due to the presence of various frequency spectrums. The EMG signal is a complex physiological signal that potentially consists of a superposition of various Motor Unit Action Potentials (MUAPs). Numerous studies have demonstrated that Time-Frequency decompositions can provide valuable insights for underlying diagnostics [27].

In this study, we chose to leverage Audio Spectrogram Transformers (AST), as they operate on the Time-Frequency representation of EMG signals (spectrograms). Additionally, in the field of Deep Learning, transfer learning has proven to be a valuable asset [28], even when the underlying deep learning model has been trained on a different modality. We found both the Time-Frequency representation and the transfer learning capabilities of Transformers to be attractive features for our study, as our results suggest.

We employed transformers that leverage attention mechanisms to analyze EMG data, aiming to classify signals into control vs. neurogenic and control vs. OSA classes. Our model, inspired by the ViT, encodes EMG signals into latent vectors. This approach is particularly novel in the context of EMG data, as Transformers are typically employed in processing 2D image data or sequential text data.

**Fig. 1:**
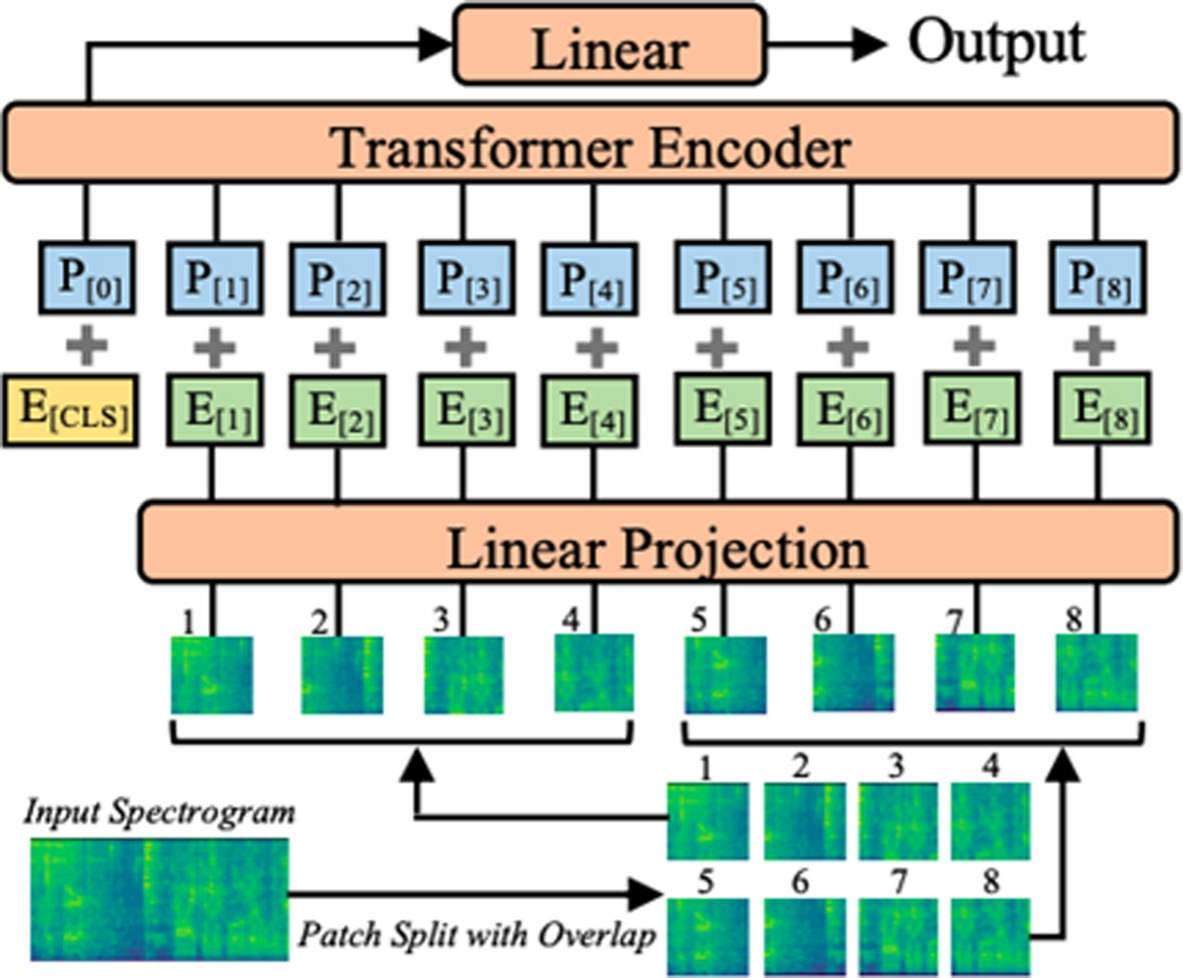
Model Architecture: Example of an Audio Spectral Transformer [23] (CC BY 4.0; arXiv:2104.01778)

Our methodology included three main strategies to train a robust model on a limited dataset:

1. **Transfer Learning**: We adapted an Audio Spectral Transformer (AST; Fig. 1), a variation of ViT, pre-trained on extensive audio datasets, to understand the spectral dynamics of EMG signals.
2. **Simulated Data Augmentation**: To counter the limited dataset size, we leveraged simulated data to pre-train the AST on 6,000 simulated EMG recordings, converting to spectrograms and using standard backpropagation for fine-tuning. Thousands of recordings were generated by validated EMG simulation [29], resulting in an array of abnormalities providing a rich training environment that enhances the model’s ability to generalize from limited real patient data to expose the algorithm to a wide variety and large sample to maximize training and accuracy.
3. **Regularization Techniques**: We implemented several regularization strategies to prevent overfitting, ensuring the model performance remains consistent across new, unseen data.

**Fig. 2:**
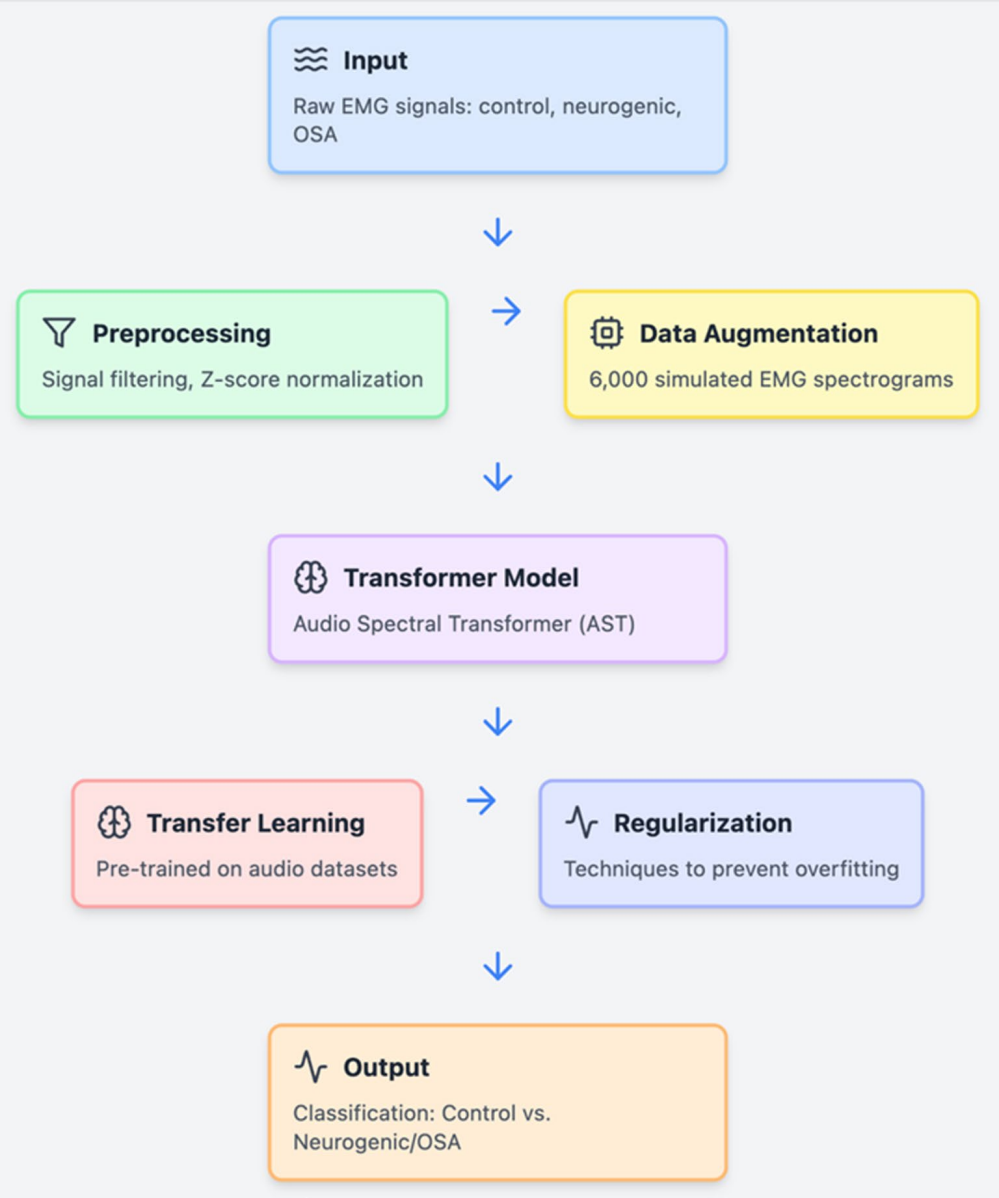
Overview of the data processing and Deep Learning model finetuning

Additionally, we incorporated advanced signal processing techniques, including filtering and normalization, to prepare the EMG data for effective Transformer learning. Signals were pre-processed to remove noise and normalized using Z-score normalization, adapting from practices common in high-density EMG signal analysis [30]​. These steps are crucial for reducing variability in signal quality and enhancing model training efficiency (Refer to Fig. 2 for an overview).

Two experiments were performed: one to assess the ability of the DL model to distinguish between control and neurogenic cases, and a second to assess the ability to distinguish between control and OSA cases. We included neurogenic cases to understand the generalizability of the DL architecture to different disease states and to assess the potential for this methodology to identify OSA patients with neuromuscular pathology. The overarching objective of this work was to develop an algorithm to distinguish between healthy and pathological EMG signals, leveraging audio spectral transformers and their proficiency in learning from sequential data with time-frequency representations. As shown in Fig. 1, tmEMG can be modeled well by sequentially analyzing overlapping time-frequency segments and identifying features across frequency bands and time.

### Classifying control vs. neurogenic cases

In each experiment, cases were tested using training, validation, and testing sets in a 4-Fold Cross-Validation Process. In each fold, one patient’s data was exclusively reserved for testing. The data of the remaining four patients were then shuffled and randomly allocated between the training and validation sets. This approach ensured that each patient contributed to the testing set exactly once, mitigating potential biases. We opted for patient-based splitting primarily to address two key concerns: data leakage and validation set importance. The segment level classification is done using a 50% probability threshold. We extrapolated this approach further to the patient level, taking an average probability over all segments and again setting this against a threshold of 50%. We did not adjust either of these thresholds to observe for potential improvement in accuracy.

#### Avoiding data leakage

If the folds were created by randomly sampling individual EMG recordings, the model might leverage patient-specific characteristics instead of focusing on the generalizable features indicative of OSA. This phenomenon, known as data leakage, could unfairly inflate the model’s performance by providing access to information it should not have during testing. Patient-based splitting effectively eliminates this risk by ensuring that the test set contains data from completely unseen individuals.

#### Validation set Importance

In deep learning models, the validation set plays a critical role in determining when to stop training. It allows us to monitor the model’s performance on unseen data during training, preventing overfitting and optimizing hyperparameters accordingly. Without a dedicated validation set, relying solely on training metrics could lead to an overly optimistic assessment of the model’s generalizability. Patient-based splitting guarantees the presence of a robust validation set in each fold, further improving the reliability of our model evaluation.

By segregating a dedicated test set, completely independent of the training and validation data, we ensure that the final assessment of our model is unbiased and reliable. This test set represents a fresh, unseen challenge for the model, providing a more accurate reflection of its true performance in real-world scenarios.

While patient-based splitting offers significant advantages, it also presents challenges. Notably, the training data size in each fold becomes limited, with only one patient’s data contributing to the test set for each class. However, we believe for the purposes of this pilot study that this trade-off is necessary to prevent data leakage and ensure the model’s ability to generalize effectively across different patients.

We averaged the predicted probabilities of each patient’s recording segments to create a combined score for each patient. This method allows for quantification of abnormality in addition to classification and requires the choice of a threshold for classification. In the future, this threshold will be adjusted to identify the optimal threshold to achieve the greatest accuracy.

Although data from needle EMG and tmEMG have been found to have equivalent diagnostic quality, we analyzed data separately as well as combined to ensure no significant differences and to further support the use of tmEMG as having equivalent quality to standard needle EMG.

### Data analysis

Performance was evaluated using accuracy, sensitivity, specificity, and F1-score metrics in a 4-fold cross-validation design. Data were analyzed as an aggregate as well as grouped by electrode type used; tmEMG™ (Powell Mansfield, Inc., San Diego, USA), or standard EMG needle electrode (Ambu, Inc., Columbia, MD, USA Neuroline 25 mm x 30G).

## **Results** – classifying control versus neurogenic cases

Using combined needle EMG and tmEMG data, the model exhibited a high sensitivity for Neurogenic cases (98%), and moderately high specificity (73%), which could be indicative of the model’s training data distribution or feature representation bias towards the Neurogenic class, which is often more rigorously characterized in clinical datasets.

The performance of the model did not show a significant difference whether using tmEMG or needle EMG data, demonstrating the same high sensitivity (98%) and moderate specificity (71–73%) for needle EMG, tmEMG, and with both combined, indicating the comparable diagnostic ability of tmEMG compared to nEMG.

Regarding classification performance across the four folds between Control and Neurogenic cases, Validation Accuracy ranged from 79.6 to 90.6% across four folds. The highest accuracy was in Fold 2 (90.6%), and the lowest in Fold 4 (79.6%). Test Accuracy varied between 81.05% and 88.42%, with the highest in Fold 4 (88.42%). Note that the test set is the same for each fold, the best model per each fold is evaluated.

**Fig. 3:**
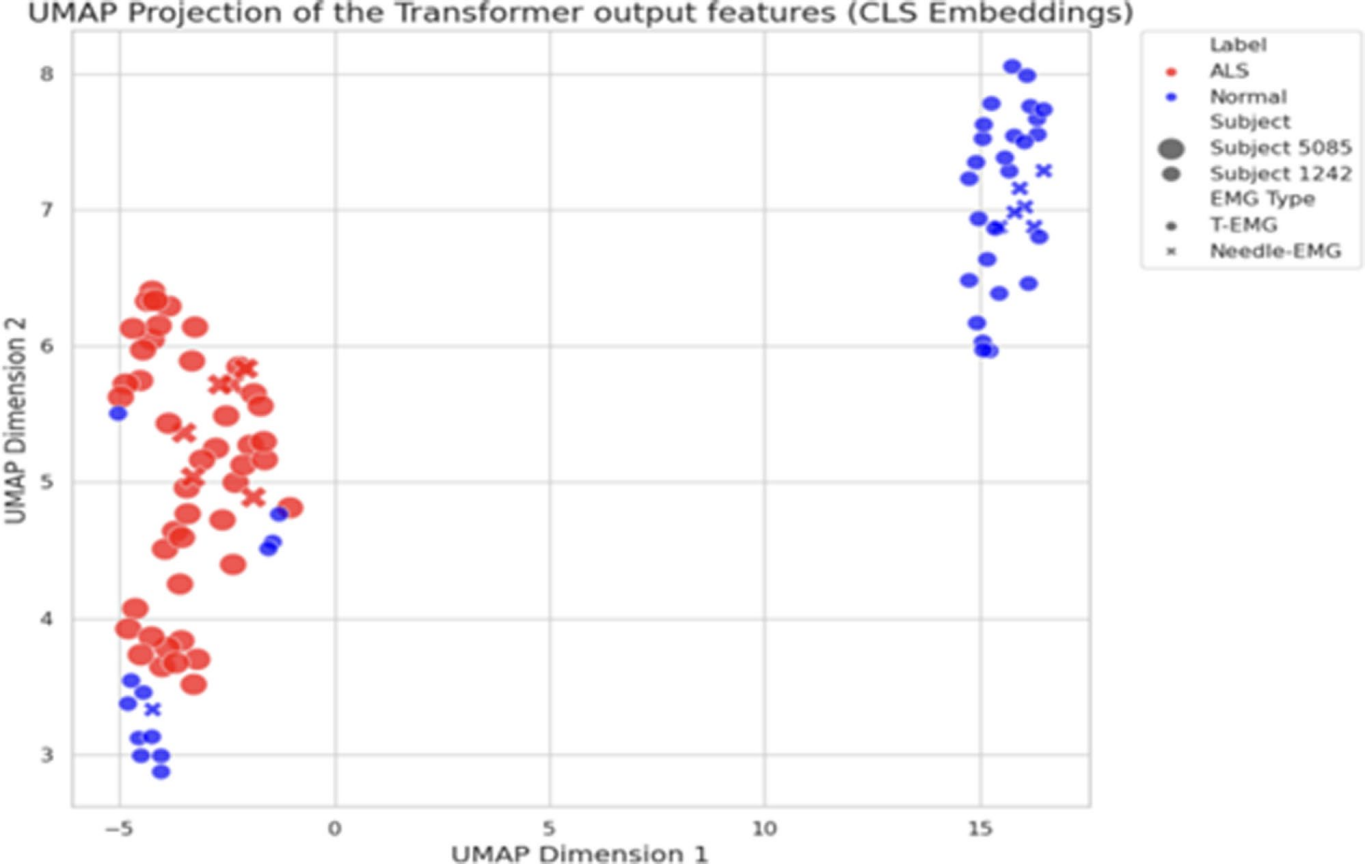
An example of Uniform Manifold Approximation and Projection (UMAP) for neurogenic versus control classification using data from one ALS patient and one healthy control. UMAP: Uniform Manifold Approximation and Projection, CLS: Classify token, ALS: Amyotrophic lateral sclerosis

The Uniform Manifold Approximation and Projection (UMAP; Fig. 3) plot offers a compelling visual representation of the Transformer model’s performance on the task of classifying control and Neurogenic EMG data. Using the two identified dimensions, it is clear that the model distinguishes well between the two patients. 

## **Results** – classifying control versus OSA cases

Using combined needle EMG and tmEMG data, the model exhibited moderately high sensitivity for OSA cases (88%), and moderate specificity (64%). The performance of the model did not show a significant difference whether using tmEMG or needle EMG data, again demonstrating similar sensitivity and specificity for needle EMG, tmEMG, and with both combined, indicating the comparable diagnostic ability of tmEMG compared to nEMG.

Table 1 depicts the classification performance across the four folds between Control and OSA cases. Validation Accuracy ranged from 52 to 73% across four folds. The highest accuracy was in Fold 4 (72%), and the lowest in Fold 1 (52%). Test Accuracy varied between 61.5% and 77%, with the highest in Fold 1 (77%). Validation F1-Score ranged from 55 to 83% across four folds, while Test F1-Score ranged from 62 to 81.8%.

**Table 1:** Performance characteristics of the transformer model on classifying control and OSA patients, grouped by results from validation and testing sets, using combined tmEMG and needle EMG data. OSA: obstructive sleep apnea

Fold	Accuracy (validations)	Accuracy (test)	F1-Score (validations)	F1-Score (test)
1	0.52	0.77	0.55	0.818
2	0.635	0.615	0.67	0.62
3	0.73	0.74	0.83	0.8
4	0.72	0.71	0.78	0.794

**Fig. 4:**
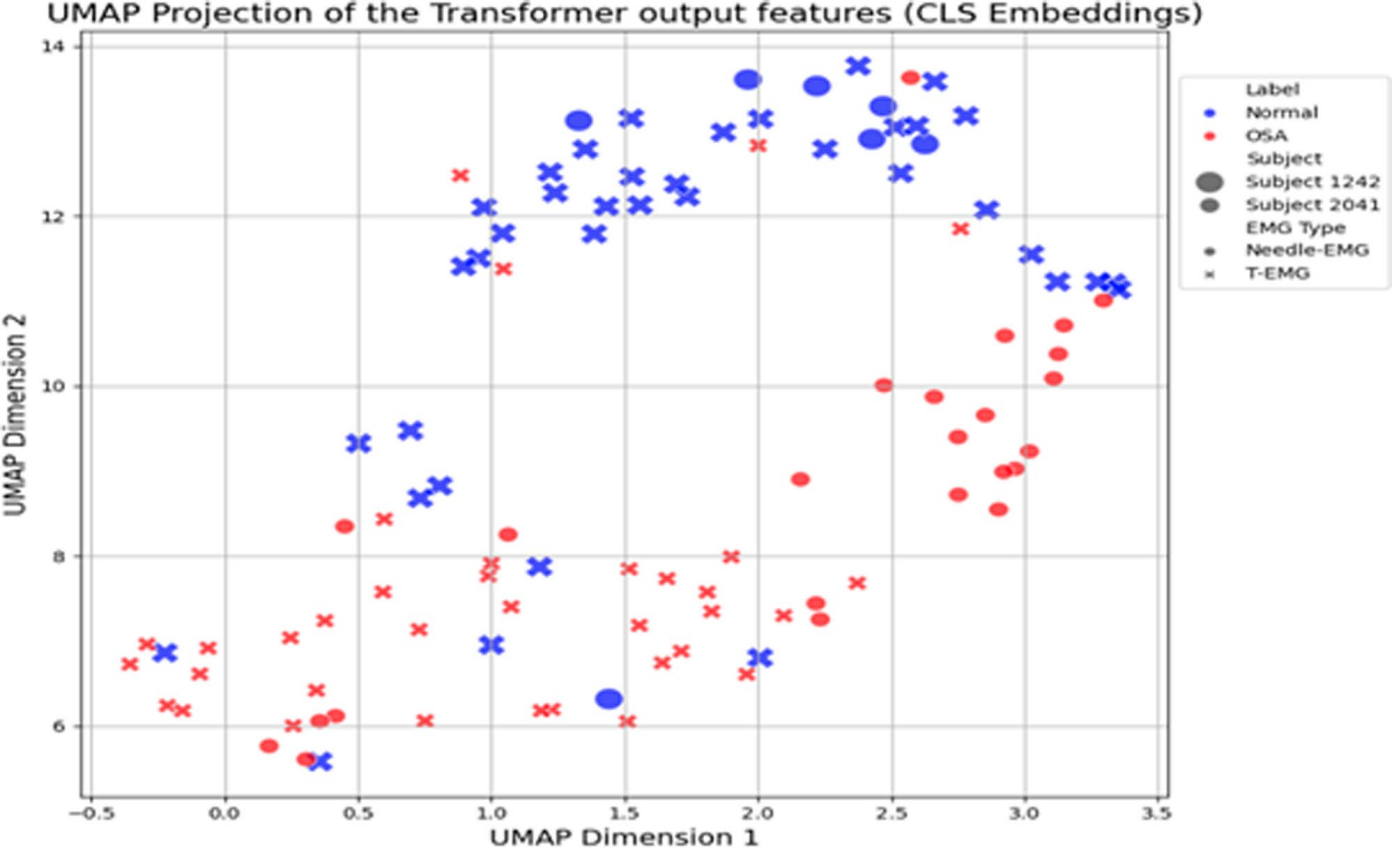
Uniform Manifold Approximation and Projection (UMAP) for OSA versus Control classification using data from one OSA patient and one healthy control. UMAP: Uniform Manifold Approximation and Projection, CLS: Classify token, OSA: obstructive sleep apnea

The UMAP plot visual representation of the Transformer model’s performance on the task of classifying Control and OSA EMG data (Fig. 4) demonstrates the Transformer model’s performance when classifying OSA and controls. The distinction between groups is not as pronounced as compared to the UMAP representation when classifying Neurogenic and controls (Fig. 3), suggesting greater electromyographic similarity between the two classes and a requirement for increased training to optimize the model’s performance.

Table 2 displays the results of averaging the predicted probabilities of each segment for individual patients across all 11 OSA and control patients, achieving an accuracy of 82% in correctly classifying the two groups.

**Table 2:** Accuracy of combined tmEMG and needle-EMG data in classifying control vs. OSA

ID	Group	Confidence for Normal	Confidence for OSA	Final Classification
1242	Control	0.66	0.34	Correct
2041	OSA	0.15	0.85	Correct
3578	Control	0.35	0.65	Misclassified
3161	OSA	0.22	0.78	Correct
2300	Control	0.63	0.37	Correct
6262	OSA	0.4	0.6	Correct
9588	Control	0.63	0.37	Correct
8435	Control	0.31	0.69	Misclassified
1537	OSA	0.11	0.89	Correct
3826	OSA	0.17	0.83	Correct
4309	Control	0.52	0.48	Correct
			**Accuracy**	**82%**

## Discussion

Diagnosis in OSA management is a well-known concern within the community [7] and causes a major healthcare disparity within the population. Prior studies have suggested a potential utility for EMG in OSA, but the field has remained nascent due to the invasive nature of the testing and the lack of experience in interpreting the signals recorded. The first of these issues was addressed recently by the design of a novel surface transmembranous probe for intra-oral EMG recordings, tmEMG. The second issue, lack of experience in interpreting intra-oral EMG from OSA patients, is the subject of this study, and together these innovations have the potential to enable the field to rapidly move forward and take advantage of this novel and significant diagnostic avenue.

To diagnose OSA, the gold standard method has traditionally been PSG, an overnight test conducted in a sleep lab [31]. PSG uses over seven channels to monitor sleep, respiration, and muscle activity, including electroencephalography, electrooculography, EMG, electrocardiography, airflow monitoring, respiratory effort, and pulse oximetry. The PSG report provides detailed information about sleep, limb movements, and the presence of sleep-disordered breathing based on the apnea-hypopnea index (AHI). Because PSG is expensive, labor and time-intensive, and lacks versatility as an on-demand pre-surgical and pre-sedation screening tool, several alternative screening and diagnostic technologies have been evaluated throughout the years to broaden access to diagnostic services.

### Alternatives to PSG in OSA diagnosis and screening

Although lacking EEG and sleep stage assessment [8], the most common alternative to PSG is the HSAT. However, several studies have pointed to issues with HSAT; false negatives may be significant in high-risk patients (18%) [32], HSAT may underestimate AHI severity by about 10% [33], specificity increases while sensitivity decreases as severity of sleep apnea increases, and technical failure is an important concern [34].

Both PSG and HSAT are unable to inform on the various mechanistic subtypes of OSA, which is essential for making personalized therapeutic decisions. Additionally, existing medical insurance paradigms do not sufficiently cover PSG or HSAT to allow for repeat testing as is needed to monitor responses to therapy. Furthermore, the cumbersome nature of each test prevents the straightforward point-of-care assessment that is crucial for reducing morbidity and mortality associated with this prevalent condition.

A few other techniques have been evaluated showing a moderate ability to diagnose OSA, including oximetry and acoustic devices. Several studies have found a correlation between oximetry (oxygen desaturation index; ODI) and PSG AHIs [35–40]. One study that has not been replicated to date did report 82% sensitivity and 94% specificity^36^; however this was in moderate to severe OSA patients and relied on oximetry from the PSG itself, suggesting incorporation bias, in addition to a selective population [39]. Acoustic devices have also been evaluated in OSA diagnosis and screening through a few exploratory studies, although only at most moderate accuracy has been reported [41]. Based on evidence to date, neither technique is recommended for the evaluation of OSA at this time [31]. Furthermore, these techniques, similar to PSG and HSAT, rely on overnight measurement, making them incompatible with the need for immediate, point-of-care testing.

Screening for OSA, in primary care, inpatient settings, and pre-surgically, has primarily focused on questionnaires, specifically Berlin, STOP, STOP-BANG, and ESS [31]. Overall, STOP-BANG appears most suitable as a screening test when considering both sensitivity and specificity; however, specificity is fairly low, especially pre-surgically, ranging in one study from 30 to 43% [42], with important implications including unnecessary testing, costs, and delays. STOP-BANG receiver operating curves (ROC) at varied OSA severity have implied that a cutoff score of 5 results in the optimal sensitivity and specificity for moderate-severe OSA (60%, 72%) and surgical populations (45%, 56%)^31^. Despite the lower specificity, the International Consensus Statement on Obstructive Sleep Apnea [31] recommends patients undergo screening and, at this time, this is best carried out using STOP-BANG. Should results of our pilot study hold up in larger studies with diverse populations, the initial estimates for sensitivity and specificity for detecting OSA of 88% and 64% compare favorably, especially to pre-surgical questionnaire performance. However, there was notable variability in model performance across different data folds, suggesting that our model might benefit from further tuning, more sophisticated data handling strategies to improve consistency, and increasing exposure to additional data and the full spectrum of EMG signal characteristics in OSA.

### Electromyography and machine learning in OSA

The potential role of EMG in OSA has been evaluated in several studies, generally involving intramuscular microwire electrodes or submental surface electrodes [8, 10, 12]. The focus has predominantly been on detecting neurogenic or myopathic signals from the genioglossus of patients with OSA, which has generally been interpreted as being caused by traumatic vibratory damage or hypoxemia, or alternatively as causative of OSA secondary to weakness of pharyngeal dilator musculature [10]. These studies have identified predominantly neurogenic pathology associated with OSA, although the relationship remains to be clarified. Few studies have commented on the utility of EMG in detecting OSA and have relied on detecting differences in resting activity in the genioglossus to differentiate healthy controls from OSA patients with moderate success [13, 15]. There was likely little traction due to a lack of advancements in probe and electrode design as well as quantitative EMG techniques.

No other studies to our knowledge have attempted to apply machine learning methodology to EMG signals recorded from the muscles of the oral cavity to detect OSA, whether using needles or surface electrodes. Although obtained using transmembranous surface recording from the genioglossus and palatoglossus, the design of the tmEMG probe and lack of intervening subcutaneous tissue results in our probe containing substantially equivalent information to the more sensitive needle EMG techniques [22]. Numerous studies have assessed the effectiveness of machine learning models in distinguishing neuromuscular pathology using EMG recordings obtained from standard clinical needle EMG of limb muscles: a recent scoping review article [43] summarizes the literature on machine learning in needle-based EMG well, reviewing 51 studies in detail. It should be noted that the majority of included studies (71%) used small open-source data sets such as the EMGLab data set [44] or a PhysioNet data set [45]. EMGLab data set consists of recordings (from the limbs, not oral cavity) of 10 healthy controls, 8 ALS patients, and 7 myopathy patients and all studies using this data set were focused on classification of EMG signal as being neurogenic, myogenic, or healthy. The PhysioNet data set used by some studies comprises only a single patient in each category (neuropathy, myopathy, and healthy controls). The majority of the studies used machine learning techniques while 8% implemented deep learning techniques. Overall, older studies reported very high accuracy but generally suffered from significant issues such as data leakage and the use of signal-level rather than patient-level split, resulting in likely overestimation of performance up to levels as high as 100% accuracy. However, more recent studies implementing deep learning techniques and several of similar sample size to this study, were found to be of higher quality (based on degree of adherence to CLAIM criteria [24]), and therefore validity, but slightly lower performance, up to around 85% accuracy. In our study, the performance for detecting neurogenic pathology in an EMG segment was found to be high, with a sensitivity and specificity of 98% and 73%, respectively. The high sensitivity for detecting ALS cases in this instance may result because the severity of the neurogenic abnormality in the ALS EMG recordings was in most cases not mild. Performance might drop with the inclusion of more borderline cases but greater exposure to the variety and full spectrum of pathology would enhance generalizability and is the focus of ongoing research.

The results of this pilot study show promise in the ability of EMG signals, obtained non-invasively using a tmEMG probe, to not only detect neuromuscular pathology (that might be related to subcategories and endotypes of OSA) but also the potential to act as a simple, cost-effective, and practical diagnostic test for the detection of OSA itself. Because the absolute numbers of patients were low (6 healthy, 5 OSA, 5 ALS patients), this will reduce generalizability of our findings despite the several important methods put in place to mitigate this, discussed above.

Of note, our findings were consistent whether nEMG or tmEMG signals were used, indicating comparable information content. Furthermore, UMAP visualization of the model’s learned representations showed that recordings from the two modalities often cluster together for controls and separately for abnormal recordings, suggesting tmEMG appears capable of effectively replacing the more invasive nEMG recordings without loss of accuracy, and supporting findings from the prior study [22]. An important method employed in this study was the use of varied simulated EMG data that included a variety of levels of simulated pathology for pretraining to maximize model accuracy. Combined with AST architecture, this approach demonstrates the potential to achieve valid results even within small sample-size datasets. This approach aligns with recent trends in machine learning, where simulated data are increasingly used to bolster models before real-world application and may allow earlier identification of promising avenues without the often-insurmountable requirement for large quantities of data and could dramatically increase the efficiency of research.

The most notable finding was that by averaging the predicted probabilities of each segment for individual patients, the model correctly classified up to 82% of subjects as belonging to either the control or OSA classes. This approach allows *quantification* of the degree of abnormality, which could be used for clinical decision-making as well as for outcomes research to minimize costly studies of new therapies coming down the pipeline.

As discussed, the limitations of this pilot study were mainly based on the small sample size, which reduces the degree of confidence in interpretation. As this is a pilot study and there was limited information regarding testing characteristics necessary for power calculations, tests of significance are not possible. To mitigate the small sample size, we leveraged transfer learning from the pre-trained AST architecture, further fine-tuned the model with large numbers of a variety of simulated EMG data and employed an optimal testing and validation methodology. Prospective studies based on larger sample sizes comparing control and OSA patients are ongoing by our group and are needed to optimize the model and assess its full diagnostic capability and generalizability.

The low-force protocol used for acquiring EMG signals in this study did not account for variations across the respiratory cycle, which might contain crucial diagnostic information. Future protocols should consider including dynamic muscle activity to capture a broader range of physiological data.

The variability in fold-specific performance metrics raises questions about the robustness of the model. Future studies could explore more diverse and larger datasets to validate the model’s efficacy and potentially develop a more robust algorithm that can perform uniformly well across different subsets of data.

In summary, our study demonstrates a potential for deep learning to enhance tmEMG to classify and quantify OSA and neurogenic patients and further underscores the excellent concordance between standard needle EMG recordings and those from the newly designed non-invasive tmEMG probe. Further work is currently underway to increase sample size to improve generalizability and confidence in the results.

## Conclusion

The application of deep learning methods to tmEMG data from muscles of the oral cavity and oropharynx shows promise not only for the characterization of neuromuscular pathology but also potentially for the diagnosis of OSA, which would have exciting implications for the current state of OSA diagnostics. However, larger prospective studies are required.

## Data Availability

Partial data is provided within the manuscript, and the remainder can be provided upon request.
